# The Impact of Earthquake-Induced Healthcare Disruption on an Established Developmental Hip Dysplasia Screening Program in an Earthquake Zone

**DOI:** 10.3390/children13081055

**Published:** 2026-08-07

**Authors:** Okan Aslantürk, Sultan Nisa Çabuk, Emre Ergen, Hüseyin Utku Özdeş, Fırat Al, Tahsin Sarıbas, Murat Kılıç, Hakan Ertem, Mustafa Karakaplan, Sevgi Demiröz Taşolar, Hüseyin Ayvaz, Aybars Kıvrak, Şenol Bekmez

**Affiliations:** 1 Department of Orthopedic and Trauma Surgery, Faculty of Medicine, Inonu University, Malatya 44280, Türkiye; 2 Faculty of Medicine, Inonu University, Malatya 44280, Türkiye; 3 Department of Pediatric Radiology, Faculty of Medicine, Inonu University, Malatya 44280, Türkiye; 4 Private Clinic, Adana 01120, Türkiye; 5 Texas Children’s Hospital, Baylor College of Medicine, Houston, TX 77030, USA; sxbekmez@texaschildrens.org

**Keywords:** hip dysplasia, hip ultrasound, natural disaster, late presentation, Pavlik harness

## Abstract

**Highlights:**

**What are the main findings?**

- A major earthquake caused an acute collapse in DDH referrals followed by a statistically significant surge in late-presenting cases concentrated in a single spring quarter one year after the disaster.
- DDH incidence among late-presenting infants was markedly elevated during the earthquake year, and Pavlik harness failure requiring closed reduction emerged in post-earthquake cases where none had occurred before.

**What is the implication of the main finding?**

- Major disasters produce bimodal disruption to DDH screening—an acute access failure followed by a delayed, temporally concentrated surge in missed cases—a pattern distinct from pandemic-related disruptions and not previously described in the earthquake literature.
- Post-disaster recovery plans should incorporate calendar-specific DDH screening recall strategies to identify unscreened infants through existing primary care systems.

**Abstract:**

**Background/Objectives**: Pandemics and natural disasters may delay the diagnosis and treatment of developmental dysplasia of the hip (DDH). In this study, we evaluated the effect of 2023 Kahramanmaraş earthquake-induced healthcare disruption on the DDH screening program. **Methods**: Medical records of infants referred for DDH screening between February 2022 and February 2025 were retrospectively reviewed. Three consecutive twelve-month intervals: the pre-earthquake year (February 2022 to February 2023), the earthquake year (February 2023 to February 2024), and the post-earthquake year (February 2024 to February 2025) were evaluated to detect early and late effects of the earthquake on the DDH screening. **Results**: A thousand infants in the pre-earthquake year, 404 in the earthquake year, and 824 in the post-earthquake year were evaluated. A 60% reduction in referrals was detected during the earthquake year. DDH incidence in late presentations during pre-earthquake, earthquake, and post-earthquake years was 18.6%, 45.8%, and 22.2%, respectively. Pairwise analysis revealed a statistically significant increase in the post-earthquake year compared with the pre-earthquake period (Yates-corrected chi-square = 4.119, *p* = 0.042). No significant difference was found between the pre-earthquake year and the earthquake year (Yates chi-square = 1.362, *p* = 0.243), nor between the post-earthquake years (Yates chi-square = 0.084, *p* = 0.772). **Conclusions**: The earthquake caused significant disruption in healthcare and DDH screening. Health authorities planning post-disaster service recovery should proactively augment DDH screening capacity for infants who missed screening during the acute phase.

## 1. Introduction

Developmental dysplasia of the hip (DDH) encompasses a spectrum of conditions ranging from mild acetabular dysplasia to frank dislocation. When detected in the first weeks of life, the majority of cases can be managed conservatively with a Pavlik harness or another type of abduction orthosis. Delayed diagnosis beyond three months significantly reduces the success of conservative treatment. Therefore, closed or open reduction would be required, which exposes the patients to general anesthesia and surgery-related complications, including avascular necrosis [1,2]. Because of this, early diagnosis with screening is essential in DDH [3]. A nationwide selective newborn hip ultrasound screening program has been implemented in Türkiye since 2013. However, it evolved to universal screening over time, and all newborns were referred for hip ultrasound screening within the first 2 months of life [4]. A recent study reported that the screening program significantly reduced the rate of major surgical bone interventions [4].

Disruptions in routine healthcare services can rapidly reverse these gains. During the COVID-19 pandemic, multiple studies documented steep declines in the number of DDH ultrasound examinations performed, which resulted in higher proportions of late-presented cases as well as increased rates of missed follow-ups [5,6,7,8,9].

Major earthquakes impose a qualitatively different form of disruption of healthcare in Türkiye. The most recent one, the Kahramanmaraş earthquakes of 6 February 2023, with consecutive magnitudes of 7.7 and 7.6, affected approximately 15 million people across 11 provinces and caused more than 50,000 deaths [10,11]. Hospitals sustained structural damage, elective outpatient services were immediately suspended, and healthcare workers were also directly affected as victims [12,13]. Resources were entirely redirected to acute trauma care. Unlike pandemic restrictions, earthquake disruption was geographically abrupt, demolished healthcare facilities, displaced families from their primary care providers, and required a prolonged recovery before routine services resumed. The impact of this devastating natural disaster on DDH screening and subsequent treatment requirements has not been studied.

The aim of this study was to evaluate the effects of healthcare disruption caused by the February 2023 Kahramanmaraş earthquakes on DDH screening activities and treatment patterns at a pediatric orthopedic reference center in a city within the damage zone, with a focus on diagnostic delays and management strategies.

## 2. Materials and Methods

This single-center study was conducted at the Department of Orthopedics and Traumatology of Inonu University Turgut Ozal Medical Center in Malatya. Ethical approval was obtained from the Inonu University Ethical Committee (2026/8939). A retrospective review was conducted on the medical records of patients referred to our institution for newborn hip ultrasound screening and late-presenting DDH between February 2022 and February 2025. The study period covered three consecutive twelve-month intervals: the pre-earthquake year (February 2022 to February 2023), the earthquake year (February 2023 to February 2024), and the post-earthquake year (February 2024 to February 2025), to evaluate the early and late effects of the earthquake on the DDH screening and treatment.

Eligible participants included infants residing within our institution’s catchment area who required DDH screening or treatment. An infant having any of the following criteria was excluded from the study: (1) underlying genetic, syndromic, or neuromuscular condition; (2) prior diagnosis and treatment in another institution; or (3) inadequate clinical or radiological documentation.

Presentation age was classified as early (younger than 3 months) or late (older than 3 months) at the time of the first clinical visit. We obtained the regional live birth data for the period from 1 January 2022 to 31 December 2024 from the Provincial Directorate of Health. Hip ultrasound examinations were performed by the same fellowship-trained, experienced pediatric radiologist using the standardized hip ultrasound technique described by Graff [14].

Data extracted from the medical records consisted of demographic information (sex and age at presentation), ultrasound findings, and the type of non-surgical or surgical treatment administered.

### Statistical Analysis

Statistical analyses were performed using SPSS version 26.0 (IBM Corp., Armonk, NY, USA). Descriptive statistics are presented as mean ± standard deviation for continuous variables and as numbers and percentages for categorical variables. Normality was assessed visually (histograms and Q-Q plots) and analytically using the Shapiro–Wilk test. A *p*-value below 0.05 was considered statistically significant.

Proportions of early and late presentations were compared across the three study periods using the Pearson chi-square test. Where expected cell frequencies fell below five—particularly in the February–May 2023 quarter—the Fisher–Freeman–Halton exact test supported by Monte Carlo simulation was applied. The Cochran–Armitage trend test was used to assess the directional change in late-presentation rates within the pre-earthquake baseline period.

To evaluate the impact of the earthquake on late-presentation rates, an Interrupted Time Series Analysis (ITSA) was performed using ordinary least squares (OLS) regression. Data were grouped into twelve consecutive three-month quarters (Q1–Q12). The regression model is as follows:

Y = β_0_ + β_1_T + β_2_D + β_3_P + ε

where Y represents the late-presentation rate per quarter; T denotes elapsed time (quarters 1–12); D is a binary indicator of earthquake exposure (0 = pre-earthquake; 1 = post-earthquake); and *p* represents time elapsed since the earthquake. The coefficients β_1_, β_2_, and β_3_ estimate the pre-earthquake baseline trend, the immediate level of change at the time of the earthquake, and the post-earthquake slope change, respectively.

To capture seasonal effects, the same calendar quarters (February–May, May–August, August–November, and November–February) were compared across the three years using chi-square or exact tests as appropriate. Monthly birth counts across the three years were compared using the Friedman test, with Bonferroni-corrected Wilcoxon signed-rank post hoc tests. With three pairwise comparisons, the corrected significance threshold was set at *p* < 0.0167.

## 3. Results

### 3.1. Study Population

A total of 2228 infants were referred to our institution across the three study periods: 1000 in the pre-earthquake year, 404 in the earthquake year, and 824 in the post-earthquake year. The 60% reduction in referrals during the earthquake year reflected the acute disruption to outpatient services caused by the disaster. Patient characteristics are summarized in Table 1.

Monthly birth counts were significantly higher in 2022 than in both 2023 (*p* = 0.001) and 2024 (*p* = 0.0005) (Friedman test, *p* = 0.00019). Birth counts in 2023 and 2024 did not differ significantly from each other (*p* = 0.027, above the Bonferroni-corrected threshold of *p* < 0.0167).

### 3.2. Late-Presentation Rates Across Study Periods

The rate of late presentation increased progressively across the three periods: 4.3% (43/1000) in the pre-earthquake year, 5.9% (24/404) in the earthquake year, and 6.55% (54/824) in the post-earthquake year. Overall comparison across all three periods did not reach statistical significance (chi-square value = 4.716, df = 2, *p* = 0.094). However, pairwise analysis revealed a statistically significant increase in the post-earthquake year compared with the pre-earthquake period (Yates-corrected chi-square = 4.119, *p* = 0.042). No significant difference was found between the pre-earthquake year and the earthquake year (Yates chi-square = 1.362, *p* = 0.243), nor between the post-earthquake years (Yates chi-square = 0.084, *p* = 0.772).

**Figure 1:**
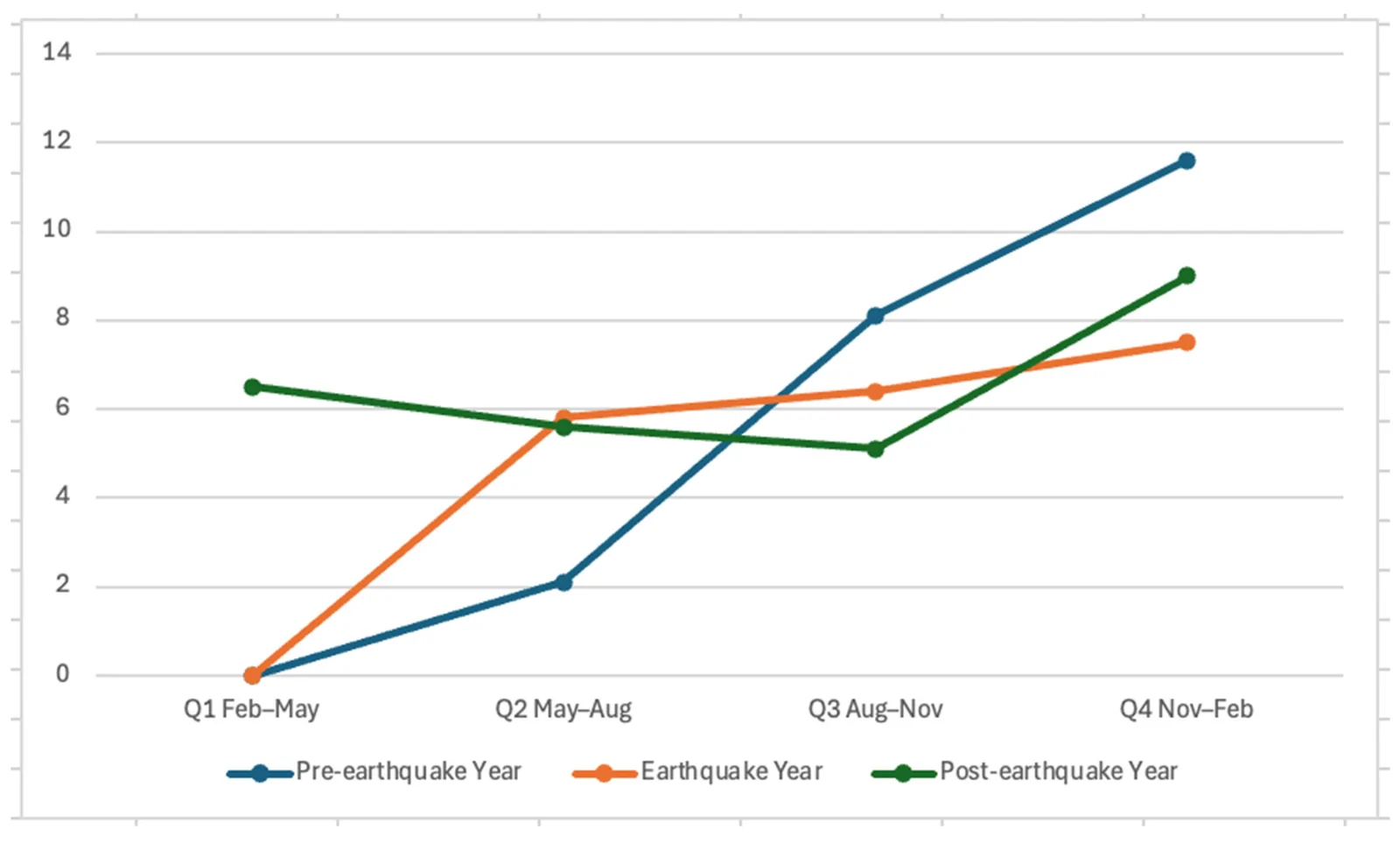
Year-over-year comparison of late-presentation rates by seasonal quarter.

To better understand the timing of late presentation after the earthquake, quarterly comparisons across the three calendar years were performed (Figure 1). A statistically significant difference was found exclusively in the February–May quarter (Q1), driven by a surge in late presentations in spring 2024 (*p* < 0.001). In this period, the late-presentation rate rose from 0% in both 2022 and 2023 to 6.5% in 2024 (11 of 168 patients). No statistically significant differences were observed across years in the May–August (*p* = 0.080), August–November (*p* = 0.452), or November–February (*p* = 0.473) quarters.

### 3.3. Interrupted Time Series and Trend Analysis Findings

To evaluate the temporal changes in late application rates during the pre-earthquake period (Q1–Q4), a Cochran–Armitage trend test was performed. The results revealed a highly significant positive upward trend in late applications over the one-year period preceding the earthquake (*p* < 0.001).

An Interrupted Time Series Analysis (ITSA) was applied to model the impact of the disaster on application patterns, capturing both acute-level changes and long-term trend interruptions. The coefficients (β) of the Ordinary Least Squares (OLS) regression model yielded the following results:

- Pre-Earthquake Underlying Trend ($\beta_{1}$): In the pre-earthquake period (Q1–Q4), a significant and consistent quarterly increase of 3.67% in late application rates was observed ($\beta_{1}=3.67$, p=0.002).
- Acute Shock and Level Change ($\beta_{2}$): During the Q5 period, when the February 6 earthquake occurred, a highly significant, abrupt drop (level change) in late application rates was observed. The disaster sharply reduced the rising application trend by 10.93%, effectively resetting the system ($\beta_{2}$ = −10.93, *p* = 0.003).
- Post-Earthquake Recovery Trend ($\beta_{3}$): In the post-earthquake period (Q5–Q12), the acceleration of late applications changed significantly, flattening relative to the steep climb before the earthquake ($\beta_{3}=-3.06$, p=0.008). During the recovery period, the system progressed toward the previous late-application volumes, with a more stable, horizontal trajectory.

### 3.4. Graf Classification and Treatment of Late Presentations

Eight of the 43 late-presented infants in the pre-earthquake year had DDH, and 6 (75%) were girls. Eleven of 24 late-presented infants in the earthquake year had DDH, and 8 (73%) were girls. Of the 54 late-presented infants in the post-earthquake year, 12 had DDH, and 9 (75%) were girls. DDH incidence in late presentations during pre-earthquake, earthquake, and post-earthquake years was 18.6%, 45.8%, and 22.2%, respectively (Table 2). DDH incidence in late presentations was statistically significantly higher in the earthquake year than in the pre-earthquake year (*p* = 0.025). There was no statistically significant difference in DDH incidence in late presentations between pre-earthquake and post-earthquake years, nor between the earthquake year and the post-earthquake year (*p* = 0.802 and *p* = 0.058, respectively). The initial treatment for all DDH patients was the Pavlik harness. All patients in the pre-earthquake year were successfully treated with a Pavlik harness. One patient in the earthquake year and two in the post-earthquake year failed with the Pavlik harness and were treated with closed reduction and spica casting.

## 4. Discussion

The main finding of this study is that healthcare disruption caused by the 2023 earthquake disrupted DDH screening in two stages. The first stage was an acute collapse in referrals, with patient volume falling by 60% in the earthquake year. The second stage, more consequential clinically, was a statistically significant rise in late-presenting cases in the post-earthquake year. The incidence of DDH among late presenters was 45.8% during the earthquake year, significantly higher than the 18.6% observed in the pre-earthquake year. Although the incidence of DDH in the post-earthquake year returned toward baseline (22.2%), the absolute number of affected infants increased due to a higher volume of late referrals, and the rate of Pavlik harness failure rose from 0% pre-earthquake to 9.1% and 16.7% in the earthquake and post-earthquake years, respectively.

The interrupted time series analysis revealed an important pre-existing trend that contextualizes these findings. In the four quarters preceding the earthquake, late-presentation rates were already rising at 3.67% per quarter, suggesting a background increase in delayed DDH detection independent of the disaster. The higher late-presentation rates observed in the autumn and winter quarters during the pre-earthquake period represent a consistent pattern in our data; however, since reasons for delayed presentation were not prospectively recorded, the underlying cause of this seasonal variation remains unclear and warrants further investigation. At the time of the earthquake, this trend was abruptly reversed by a sharp change, reflecting a near-complete collapse of outpatient DDH screening as the healthcare system redirected all resources to trauma care [11,12]. In the post-earthquake recovery period, the slope of increase flattened significantly relative to the pre-earthquake trajectory, indicating that the system did not simply resume its prior trajectory but entered a prolonged period of suppressed screening capacity before the delayed case burden became apparent.

The COVID-19 pandemic remains the closest available reference for large-scale DDH screening disruption. Mert Doğan and Aslantürk reported a 63% reduction in DDH ultrasound examinations during the first restriction period, with a significant increase in pathological hips among late-presenting infants in the post-restriction rebound [5]. Ayaz documented a 45.3% decline in examinations over 14 months, with a nearly twofold rise in follow-up non-compliance among infants with abnormal findings [6]. Guindani and De Pellegrin reported 28 late-diagnosed cases during and after a lockdown period, with a threefold increase in the rate of pathological hip types compared with the equivalent period in the preceding year [8]. In all of these reports, delayed cases accumulated gradually and diffusely over several months. Our data show a different pattern: the delayed case burden was sharply concentrated in a single spring quarter one year after the index event, and the dramatically elevated DDH incidence among earthquake-year late presenters (45.8%) points to a qualitatively more severe disruption than that described in pandemic-era cohorts, where pathological rates in delayed presentations were not reported to reach comparable levels.

Ömeroğlu et al. demonstrated that Pavlik harness success rates fall from 93% in infants under three months to 37% in those older than five months, with Graf type III and IV hips achieving conservative reduction in only 26% and 50% of cases, respectively [3]. Mulpuri et al. found that infants presenting after three months were more than five times more likely to have an irreducible dislocation than early presenters, requiring more complex surgical management [2]. In our cohort, all pre-earthquake late presenters were successfully managed with a Pavlik harness, but one patient in the earthquake year and two in the post-earthquake year required closed reduction and spica casting after harness failure. While the absolute numbers are small, this shift from 0% surgical escalation to rates of 8–17% across post-earthquake periods is consistent with the outcome data cited above and likely reflects the higher proportion of older, more severely dysplastic infants presenting during and after the disaster period.

These findings are particularly significant given the progress achieved by the national newborn DDH screening program. Kapıcıoğlu et al. showed that major surgical interventions for DDH fell by 72% nationally between 2015 and 2020 following the introduction of nationwide screening, with minor interventions increasing proportionally [4]. A disaster that suppresses referrals for a full year and elevates late-presentation DDH incidence to more than twice the baseline rate directly threatens these gains in the affected region. The concurrent reduction in birth counts—significantly lower in both 2023 and 2024 compared with 2022—means that the absolute at-risk population was already smaller, making the proportional rise in late presentations and pathological diagnoses even more notable. Post-disaster birth rate suppression may create a misleading impression of reduced pediatric demand while masking an accumulating backlog of unscreened infants who will require more complex care as the population stabilizes [13].

Several limitations should be acknowledged. The most important limitations are the retrospective design and data collected from a single center. But our center is a referral center for DDH cases, especially with pathological hips. ITSA was based on twelve quarterly data points, which may have obscured shorter-term fluctuations within each phase. The near-complete service collapse in Q5—with only 37 patients and zero late presentations—created a floor effect that limited statistical power for that quarter; the 0% rate reflects service collapse rather than a true clinical absence of delayed cases and was addressed using Monte Carlo-supported exact tests. The number of late-presenting DDH patients in each period is small, which limits the statistical power of treatment outcome comparisons. This is a single-center study from a tertiary referral hospital in an earthquake-affected zone, and findings may not generalize to primary care settings or less severely affected regions.

## 5. Conclusions

In conclusion, the 2023 earthquake-induced healthcare disruption caused a bimodal and clinically significant disruption to DDH screening: an immediate collapse in referrals followed by a statistically significant surge in late-presenting cases concentrated in a single spring quarter one year later, accompanied by a more than twofold increase in DDH incidence among late presenters and an emergence of cases requiring surgical escalation beyond the Pavlik harness. Health authorities planning post-disaster service recovery should proactively augment DDH screening capacity with particular attention to infants who missed screening during the acute phase and who are likely to present with more advanced pathology. A warning system can be added to the primary care physicians’ system to contact families about missed DDH screening, just as they are notified when a baby misses vaccination.

## Figures and Tables

**Table 1:** Patient characteristics by study period.

	Pre-Earthquake February 2022–February 2023	Earthquake Year February 2023–February 2024	Post-Earthquake Year February 2024–February 2025
Birth numbers	10,853	6968	8262
Infants presented for DDH screening	1000	404	824
Early presentation (<3 mo), *n* (%)	957 (95.7%)	380 (94.1%)	770 (93.5%)
Late presentation (≥3 mo), *n* (%)	43 (4.3%)	24 (5.9%)	54 (6.55%)

**Table 2:** Graf classification and treatment of late presentations.

	Pre-Earthquake February 2022–February 2023	Earthquake Year February 2023–February 2024	Post-Earthquake Year February 2024–February 2025
Late presentations	43	24	54
Graf classification			
Type I	35	13	42
Type IIB	8	10	10
Type IIC	-	1	2
Type D	-	-	-
Type III	-	-	-
Type IV	-	-	-
Treatment			
Pavlik harness	8	10	10
Closed reduction and spica casting	-	1	2

## Data Availability

The data presented in this study are available on request from the corresponding author due to privacy or ethical restriction.
